# Suppressed Histone H3 Lysine 18 Acetylation Is Involved in Arsenic-Induced Liver Fibrosis in Rats by Triggering the Dedifferentiation of Liver Sinusoidal Endothelial Cells

**DOI:** 10.3390/toxics11110928

**Published:** 2023-11-13

**Authors:** Fang Hu, Xingcheng Zhou, Qianqian Peng, Lu Ma

**Affiliations:** 1 The Key Laboratory of Environmental Pollution Monitoring and Disease Control, Ministry of Education, Department of Toxicology, School of Public Health, Guizhou Medical University, Guiyang 550025, China; hufang0519@163.com (F.H.); z18217036042@163.com (X.Z.); 17885474706@163.com (Q.P.); 2 Collaborative Innovation Center for Prevention and Control of Endemic and Ethnic Regional Diseases Co-Constructed by the Province and Ministry, Guizhou Medical University, Guiyang 550025, China

**Keywords:** arsenic, liver fibrosis, liver sinusoidal endothelial cells, H3K18ac

## Abstract

Arsenic pollution is a global environmental concern. Arsenic-induced chronic liver injury and its irreversible outcomes, including liver cirrhosis and liver cancer, threaten the health of residents in arsenic-contaminated areas. Liver fibrosis is a reversible pathological stage in the progression of arsenic-induced chronic liver injury to cirrhosis and liver cancer. The aim of this study is to identify the epigenetic mechanism of arsenic-induced liver fibrosis based on the dedifferentiation of liver sinusoidal endothelial cells (LSECs). Rats were treated with 0.0, 2.5, 5.0, or 10.0 mg/kg sodium arsenite for 36 weeks. Marked fibrotic phenotypes were observed in the rat livers, manifested by hepatic stellate cell activation and an increased extracellular matrix, as well as the deposition of collagen fibers. The reduced fenestrations on the cells’ surface and the increased expression of the dedifferentiation marker CD31 corroborated the LSECs’ dedifferentiation in the liver tissue, which was also found to be significantly associated with fibrotic phenotypes. We further revealed that arsenic exposure could inhibit the enrichment of histone H3 lysine 18 acetylation (H3K18ac) in the promoters of *Fcgr2b* and *Lyve1*, two key genes responsible for maintaining the differentiation phenotype of LSECs. This inhibition subsequently suppressed the genes’ expression, promoting LSEC dedifferentiation and subsequent liver fibrosis. In conclusion, arsenic can trigger liver fibrosis by inhibiting H3K18ac-dependent maintenance of LSEC differentiation. These findings uncover a novel mechanism of arsenic-induced liver fibrosis based on a new insight into epigenetically dependent LSEC dedifferentiation.

## 1. Introduction

Arsenic, a human class I carcinogen, is released into the environment through natural pathways, such as rock weathering, volcanic eruptions, and anthropogenic activities, such as mining, smelting, and fuel combustion. Anthropogenic activities contribute more to the presence of arsenic in environmental media than the natural pathway [1]. It has been reported that around 200 million people worldwide are threatened by unsafe arsenic levels through contaminated drinking water, food, and air [2]. Chronic exposure to arsenic has been demonstrated to induce multi-organ damage and even tumors [3]. The liver is one of the principal toxic hosts of arsenic [4]. The epidemiological data show that chronic arsenic exposure can cause abnormal liver function, fibrosis, cirrhosis, and liver cancer [5,6,7,8]. Among them, clinical and morphological studies have found liver fibrosis to be an important morphological lesion associated with chronic arsenic toxicity [9,10,11]. Liver fibrosis is a reversible pathological stage in the progression of liver injury and chronic liver disease to cirrhosis and liver cancer, and it is critical to prevent the adverse outcomes of chronic liver disease during this period [12]. However, the mechanism for arsenic-induced liver fibrosis remains unclear, which is detrimental to the early prevention and treatment of arsenic-induced liver damage.

Liver sinusoidal endothelial cells (LSECs) are highly specialized endothelial cells located in the liver sinusoids that are characterized by the presence of fenestrae in sieve plates and without a basement membrane [13]. Based on this specific fenestra structure, LSECs play a critical role in maintaining the homeostasis of the liver sinusoidal microenvironment by clearing antigens and cell debris, promoting metabolism and material exchange, and regulating immune balance [14]. The LSECs will be dedifferentiated due to prolonged damage or stimulation, which manifests as the loss of fenestra and then the absence of the gatekeeper function of the hepatic sinusoidal microenvironment [15]. It is well known that the activation of hepatic stellate cells (HSCs), which initially occurs in the pathogenesis of liver fibrosis, produces the extracellular matrix and leads to the deposition of liver collagen fibers [12]. Growing evidence has found that dedifferentiated LSECs could promote HSC activation in a cell co-culture system, which might be due to the absence of the gatekeeper function of dedifferentiated LSECs and the increased secretion of HSC activating factors, such as inflammatory factors and chemokines [16,17]. In addition, emerging evidence suggests that maintaining or restoring the differentiation phenotype of LSECs can result in the quiescence of HSCs and the regression of fibrosis [18,19]. These results suggest that LSEC dedifferentiation is a key event that promotes the activation of hepatic stellate cells and liver fibrosis. However, it is currently unknown whether LSEC dedifferentiation is involved in arsenic-induced liver fibrosis and how arsenic exposure induces LSEC dedifferentiation.

The acetylation of histone lysine residues is an important epigenetic modification that regulates the transcriptional activation of genes. Aberrant levels of histone acetylation are involved in adverse phenotype-triggering and diseases by affecting how genes are transcribed. Histone acetylation levels are regulated by both histone acetyltransferases and histone deacetylases [20]. It has been found that the specific deletion of histone acetyltransferases CBP/p300 in LSECs can lead to the dedifferentiation of LSECs and subsequent liver fibrosis [21]. The use of largazole, a histone deacetylase inhibitor, to rescue the histone acetylation level can inhibit the CCl_4_-induced dedifferentiation of LSECs and liver fibrosis [22]. These results suggest that aberrant levels of histone acetylation may be critical to triggering LSEC dedifferentiation, but the specific sites of histone acetylation that regulate LSEC dedifferentiation are still unclear.

We previously identified a specific site of histone modification in response to arsenic exposure, histone H3 lysine 18 acetylation (H3K18ac), and demonstrated its involvement in arsenic-induced oxidative damage and liver injury [23,24]. This suggests that H3K18ac may be a key driver of arsenic-triggered adverse outcomes. Additionally, recent studies have found that H3K18ac is a potential reliable target for the pathological mechanism, prognosis, and treatment of various liver diseases [25,26]. Therefore, rats were chronically exposed to sodium arsenite (NaAsO_2_) to explore the relationship between H3K18ac, LSEC dedifferentiation, and arsenic-induced liver fibrosis. In addition, H3K18ac was used to explore the mechanism of arsenic-induced LSEC dedifferentiation and its contribution to liver fibrosis to provide new evidence for identifying the mechanism and therapeutic targets of liver fibrosis induced by arsenic.

## 2. Materials and Methods

### 2.1. Animal Model

Twenty-four healthy, clean Sprague Dawley rats aged from 6 to 7 weeks, with an initial weight of 100 ± 20 g, were purchased from Liaoning Changsheng Biotechnology Co., LTD (Benxi, China) (Qualification certificate No. SCXK (Liao) [2020-0001]). All the animals were fed with a standard diet in a 20–25 °C room with a humidity of 50–60% for 12 h each day and night. After one week of adaptive feeding, the animals were divided into four groups of six animals, with a male-to-female ratio of 1:1. The rats were housed in each group, with the males and females housed separately. According to the literature [27,28], the oral median lethal dose (LD_50_) of NaAsO_2_ is 41.0 mg/kg in rats. Following the principle of sub-chronic toxicity experiments and combined with our preliminary experiments as well as previously published research [29], the NaAsO_2_ treatment groups were given 0.0, 2.5 (1/16LD_50_), 5.0 (1/8LD_50_), or 10.0 (1/4LD_50_) mg/kg NaAsO_2_ (Sigma, UA) using a gavage, respectively. The dosages of NaAsO_2_ are equivalent to 388.82 μg/L, 777.64 μg/L, and 1555.28 μg/L elemental arsenic concentrations in groundwater, respectively. These doses are in line with the reported arsenic concentrations in groundwater in arsenic-contaminated areas (50–1320 μg/L in most areas, even up to 5000 μg/L in some areas) [30]. They also covered the common range of arsenic concentrations in drinking water (300 μg/L to 900 μg/L) that cause liver fibrosis, cirrhosis, and liver cancer [10,31,32]. All the animals were treated once a day, six days a week for 36 weeks. The rats were weighed at the end of treatment before being sacrificed. A total of 1% sodium pentobarbital was used to anesthetize the rats via intraperitoneal injection. The liver tissues of the rats were perfused in situ with 37 °C normal saline containing 50 U heparin sodium until the tissue changed to a clay color. Then, the liver tissues were separated and weighed, and the rats were sacrificed. Some of the isolated liver tissues were immersed in 4% paraformaldehyde and 2.5% glutaraldehyde for liver histopathological examination and electron microscopy, respectively, and the rest were frozen at −80 °C. These animal experiments were approved by the Ethical Review Committee of Guizhou Medical University (No. 1702146) and followed the National Research Council’s Guide for the Care and Use of Laboratory Animals.

### 2.2. Analysis of Arsenic Load in Livers

After two hours of immersion in concentrated HNO_3_ + 30%H_2_O_2_, the liver tissues were put into a microwave digestion system (Milestone ETH, Mordena, Italy) at 120 °C for 5 min, 180 °C for 10 min, and 190 °C for 15 min. The content of arsenic was measured via inductively coupled plasma mass spectrometry (ICP-MS, PerkinElmer NexION 2000, Waltham, Massachusetts, USA) using digested and deacidified samples. The concentration of arsenic in the livers was quantitatively calculated using a standard curve and was standardized using digested liver tissue weights.

### 2.3. Liver Histopathology

The liver tissues fixed with 4% paraformaldehyde were embedded in paraffin, routinely dehydrated, sectioned, and attached to an anti-shedding slide. Then, they were baked in an oven at 65 °C for 2 h before staining. Hematoxylin–eosin staining was used to observe pathological changes in the liver tissues. Masson’s trichrome stain was used to evaluate collagen deposition in the liver tissues, and Image J software Pro Plus 6.0 was used to calculate the collagen volume fraction (CVF, %) to measure the content of collagen fibers. Sirius red staining combined with polarized light scanning was performed to calculate the content of collagen Ⅰ, a major collagen component in fibrous tissue.

### 2.4. Observation of LSECs’ Fenestrations in Liver Tissues

The liver tissues perfused in situ with 37 °C normal saline containing 50 U heparin sodium until they developed a clay color were fixed with 2.5% glutaraldehyde at 4 °C for 2 h and dehydrated with gradient tert-butyl alcohol. The samples were then sputter-coated with gold for 30 s using an ion sputter (Hitachi, MC1000) after drying with a critical point dryer (Quorum, K850). Fenestrations in the sinusoidal endothelial region of liver tissues were imaged using a scanning electron microscope (Hitachi, SU8100).

### 2.5. Analysis of Extracellular Matrix (ECM) Levels in Liver Tissues

A total of 50 mg of liver tissues was ground into a liver homogenate with precooled normal saline at a mass-to-volume ratio of 1:9 (g/mL), and then the supernatant was separated via centrifuging at 600 g/min for 20 min. The levels of ECM in the supernatant, including procollagen III (PN IIIP), collagen IV (COL-IV), laminin (LN), and hyaluronidase (HA), were measured according to the instructions for enzyme-linked immunosorbent assay kits (Medical Discovery Leader, Beijing, China). The ECM levels in the liver were calculated using a standard curve and normalized by liver tissue weight.

### 2.6. Analysis of Total Levels of H3K18ac Modification in Liver Tissues and the Chromatin Immunoprecipitation (ChIP) Assay

The total levels of H3K18ac modification were measured using a sandwich enzyme-linked immunosorbent assay (ELISA). A ChIP-qPCR assay was performed to measure the enrichment of H3K18ac in promotor regions of genes according to the SimpleChIP^®^ Plus Enzymatic Chromatin IP Kit (Cell Signaling Technology, Boston, MA, USA). Primers are shown in Table S1. The results of negative control IgG enrichment are shown in Figure S1. The detailed experimental procedure has been described in a previous study [23].

### 2.7. Immunohistochemistry

The expression levels of CD31 and α-SMA (Abcame, Cambridge, MA, USA) in the liver tissues were measured via immunohistochemistry. Antigen repair was performed by heating the deparaffinized sections in citrate buffer. After blocking for 30 min, the sections were incubated with a primary antibody at 4 °C overnight and a secondary antibody at room temperature for 1 h, stained with DAB for 5 min, and counterstained with hematoxylin. After dehydration and a transparency procedure, the slices were sealed with neutral gum. Five field images/each section were acquired with a light microscope (Nikon, Tokyo, Japan). Image J software was used to scan and calculate the average optical density value of CD31 or α-SMA positive expression in the images.

### 2.8. Quantitative Real-Time PCR

The total RNA isolated from the liver tissues was used for reverse transcription according to the Prime Script ^TM^ RT reagent Kit (Thermo Fisher, Waltham, MA, USA), followed by quantitative real-time PCR to assess the mRNA levels of the target genes using the TBGreen Premix Ex Taq^TM^ II Kit (Takara Bio, Tokyo, Japan). The primers are shown in Table S2.

### 2.9. Western Blot

Gels with appropriate concentrations were selected for electrophoresis according to the molecular weight of the target proteins, FCGR2B and LYVE1. Then, the gels were transferred, blocked, and incubated with FCGR2B (Immunoway, Plano, Texas, USA) and LYVE1 antibody (Affinity, Pottstown, PA, USA) at 4 °C and shaken overnight with a secondary antibody (Proteintech, Wuhan, China) at room temperature for 1 h. Chemiluminescence was performed after the exposure solution was added. Image J software was used to calculate the gray value of the target protein band to evaluate the expression level of the protein.

### 2.10. Statistical Analysis

SPSS version 23.0 was used to perform statistical analysis. One-way analyses of variance (one-way ANOVAs) were conducted to analyze the variations in indicators across multiple treatment groups, followed by pairwise comparisons between multiple groups using Bonferroni correction. The Pearson correlation analysis and correlation matrix were employed to analyze the correlation between two indicators and multiple indicators, respectively. The significance threshold was set at a *p* value of less than 0.05 (*p* value < 0.05), which was considered to indicate statistical significance.

## 3. Results

### 3.1. Chronic Arsenic Exposure Caused Liver Fibrosis in the Rats

**Figure 1:**
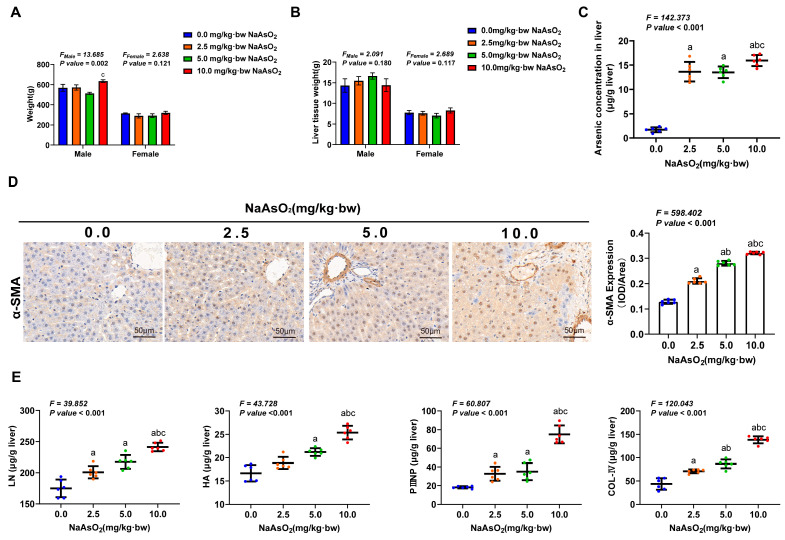
Chronic arsenic exposure induced hepatic stellate cell activation (HSC) in the rats. (**A**) Body weight, (**B**) liver weight, and (**C**) arsenic concentration in the liver were measured. (**D**) The expression of α-SMA in the livers, a marker of HSC activation, was measured via immunohistochemistry, and the average optical density of positive expression was quantitatively analyzed. (**E**) The contents of extracellular matrix (ECM) secreted by activated HSCs, including hyaluronidase (HA), laminin (LN), procollagen III (PN IIIP), and collagen IV (COL-IV), were measured via ELISA in liver tissues. The statistic “*F*” is derived from a one-way analysis of variance (one-way ANOVA). The notations “a, b, c” signify that there are significant differences (*p* value *<* 0.05) when compared with the three groups from 0.0 mg/kg to 5.0 mg/kg using Bonferroni correction, respectively.

**Figure 2:**
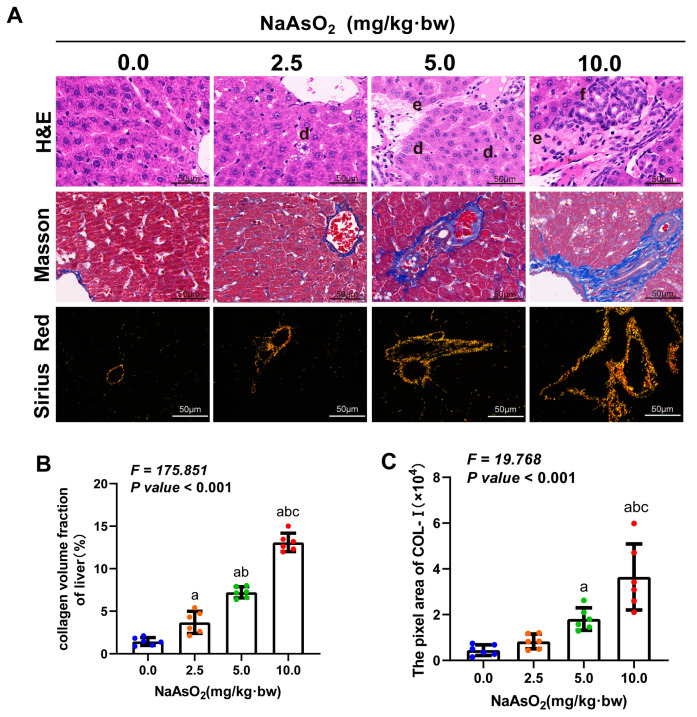
Chronic arsenic exposure induced liver fibrosis in the rats. (**A**) H&E staining, Masson’s trichrome stain, and Sirius red staining were performed to measure the histopathological changes, the area of collagen deposition, and the content of collagen Ⅰ (Col Ⅰ) (the main component of collagen fibers) in the livers, respectively. The notations “d, e, f “represent steatosis, collagen deposition, and inflammatory infiltration, respectively. The positive areas of (**B**) Masson’s trichrome stain (color in blue) and (**C**) Sirius red staining (color in yellow) were quantified. The statistic “*F*” is derived from a one-way analysis of variance (one-way ANOVA). The notations “a, b, c” signify that there are significant differences (*p* value < 0.05) when compared with the three groups from 0.0 mg/kg to 5.0 mg/kg using Bonferroni correction, respectively.

The rats were treated with a gradient dose of NaAsO_2_ for 36 weeks. We only observed a significant increase in the body weight among the male rats in the 10.0 mg/kg NaAsO_2_ group, which was compared to that of the 5.0 mg/kg NaAsO_2_ group, except that no significant differences in body weight and liver weight were observed among the groups (Figure 1A,B). Increased arsenic concentrations in the livers were found with increasing doses of NaAsO_2_ (Figure 1C). Hepatic stellate cell (HSC) activation is a well-known hallmark event of liver fibrosis. So, α-smooth muscle actin (α-SMA) expression and extracellular matrix (ECM) synthesis, the markers of HSC activation, were measured to initially evaluate whether liver fibrosis occurred in arsenic-exposed rats [33]. The results showed that the level of α-SMA expression and the contents of ECM (including procollagen III (PN IIIP), collagen IV (COL-IV), laminin (LN), and hyaluronidase (HA)) in the rat livers were increased with increasing doses of NaAsO_2_ (Figure 1D,E) and was positively correlated with the arsenic concentration in the livers (Figure S2A,B). These results suggest the activation of hepatic stellate cells in the liver tissues of arsenic-exposed rats. The results of HE staining further revealed the pathological characteristics of liver steatosis and fibrous scar in a portal area induced by arsenic exposure in the rats (Figure 2A). Masson’s trichrome stain and Sirius red staining were used to measure the area of collagen fibers deposition and the content of collagen Ⅰ (Col Ⅰ) (the main component of collagen fibers) in the rat livers, respectively (Figure 2A), and these two indicators were found to increase with an increasing arsenic dose (Figure 2B,C) and liver arsenic load (Figure S2C,D). These results suggested that arsenic exposure could induce HSC activation and liver fibrosis in the rats.

### 3.2. Dedifferentiation of Liver Sinusoidal Endothelial Cells Was Involved in Liver Fibrosis Induced by Arsenic in the Rats

**Figure 3:**
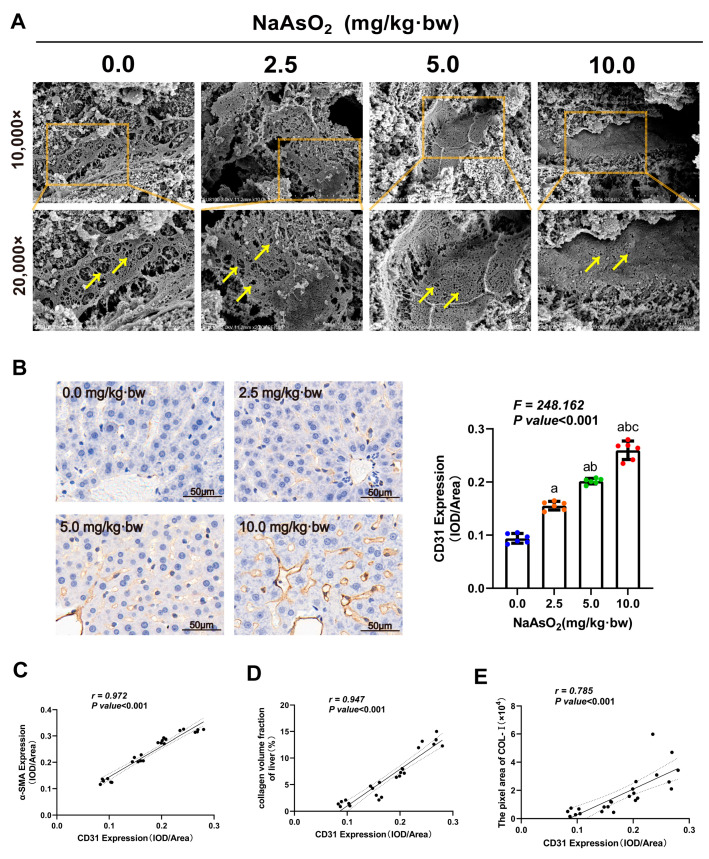
The dedifferentiation of liver sinusoidal endothelial cells (LSECs) in arsenic-induced liver fibrosis. (**A**) Fenestrations on sinusoidal endothelium of rat liver tissues were measured via scanning electron microscopy. Yellow arrows represent fenestrations on the hepatic sinusoidal endothelial surface. (**B**) Expression of CD31 in the liver, a marker of LSEC dedifferentiation, was measured via immunohistochemistry, and the average optical density of positive expression was quantitatively analyzed. The statistic “*F*” is derived from a one-way analysis of variance (one-way ANOVA). The notations “a, b, c” signify that there are significant differences (*p* value *<* 0.05) when compared with the three groups from 0.0 mg/kg to 5.0 mg/kg using Bonferroni correction, respectively. (**C**–**E**) The Pearson correlation analysis was used to assess the correlations between CD31 and the HSC activation marker (α-SMA expression), the area of collagen deposition, and the content of Col Ⅰ in the livers.

The dedifferentiation of liver sinusoidal endothelial cells (LSECs) has been found to play a critical role in triggering HSC activation [34]. To explore the relationship between LSEC dedifferentiation and arsenic-induced liver fibrosis, scanning electron microscopy was used to measure a typical feature of LSEC dedifferentiation, namely the loss of fenestrations on the cellular surface. As shown in Figure 3A, different sizes of fenestrations were observed on the sinusoidal endothelium of the rat liver tissues in the control group; on the contrary, the area and number of fenestrations on the sinusoidal endothelium in the arsenic treatment groups decreased with an increasing arsenic dose and gradually formed a basement membrane structure without fenestrations. CD31 is the marker protein of the endothelial basement membrane. The positive expression of CD31 signals LSEC dedifferentiation to form basement membrane structures. In this study, the immunohistochemistry results showed that the CD31-positive area in the hepatic sinusoidal region of the rat liver tissues increased in a dose-dependent manner with raised NaAsO_2_ doses (Figure 3B) and arsenic loads (Figure S3A). In addition, a large CD31-positive area was also observed to significantly correlate with elevated levels of α-SMA (Figure 3C) and ECM (Figure S3B) and is associated with collagen deposition and the content of Col Ⅰ (Figure 3D,E). These results suggested the involvement of dedifferentiation of LSECs in arsenic-induced hepatic stellate cell activation and liver fibrosis.

### 3.3. Inhibition of H3K18ac Was Associated with Arsenic-Induced Dedifferentiation of LSECs and Subsequent Liver Fibrosis

**Figure 4:**
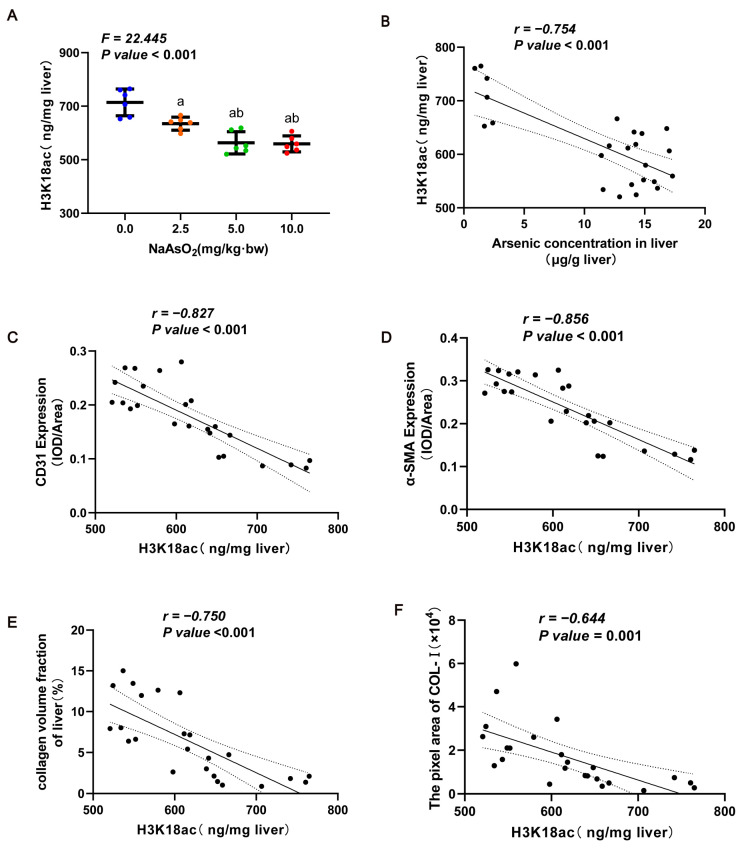
K18ac with arsenic-induced LSEC differentiation and subsequent liver fibrosis. (**A**) The total level of H3K18ac modification in liver tissue was measured via ELISA. The statistic “*F*” is derived from a one-way analysis of variance (one-way ANOVA). The notations “a, b” signify that there are significant differences (*p* value *<* 0.05) when compared with the three groups from 0.0 mg/kg to 5.0 mg/kg using Bonferroni correction, respectively. In (**B**–**F**), the Pearson correlation analysis was used to evaluate (**B**) the association of H3K18ac modification and the arsenic concentration in livers and to analyze (**C**–**F**) the relationships between H3K18ac modification and the LSEC dedifferentiation indicator (CD31 expression), the HSC activation marker (α-SMA expression), the area of collagen deposition, and the content of Col Ⅰ in the livers.

To identify the role of H3K18ac in the arsenic-induced dedifferentiation of LSECs and subsequent liver fibrosis, ELISA was performed to measure the total level of H3K18ac modification in the liver tissues. The results found that the level of H3K18ac modification in the livers was reduced with increasing doses of NaAsO_2_ (Figure 4A) and was significantly associated with an increased liver arsenic load (Figure 4B). In addition, the inhibition of H3K18ac modification was negatively correlated with the increased expression of CD31 (Figure 4C) and corresponded to elevated levels of α-SMA (Figure 4D) and ECM (Figure S4), as well as increased collagen deposition and Col Ⅰ contents (Figure 4E,F). Based on these findings, LSEC dedifferentiation is involved in arsenic-induced liver fibrosis; H3K18ac might play an important role in arsenic-induced LSEC dedifferentiation and subsequent liver fibrosis.

### 3.4. Repressed H3K18ac Regulated Arsenic-Induced Dedifferentiation of LSECs by Inhibiting Transcription of Specific Genes That Maintain Differentiation Phenotypes

**Figure 5:**
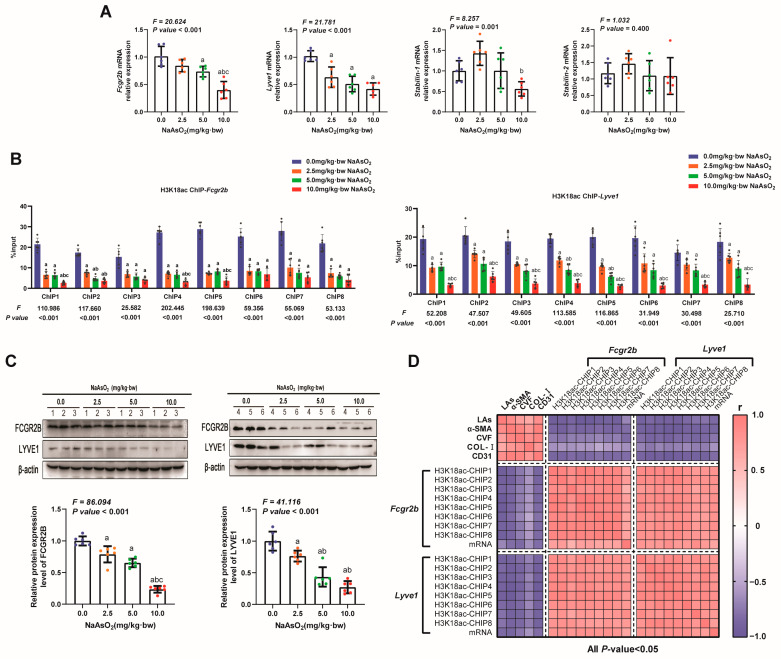
Repressed H3K18ac regulated arsenic-induced dedifferentiation of LSECs by inhibiting specific genes that maintain differentiation phenotypes. (**A**) The mRNA levels of specific genes that maintain the differentiation phenotype of LSECs in rat livers responded to NaAsO_2_ treatment, including immunoglobulin gamma complex receptor IIB (*Fcgr2b*), lymphatic endothelial hyaluronic acid receptor 1(*Lyve1*), and liver sinusoidal scavenger receptors Stabilin-1 and Stabilin-2. (**B**) The amount of enrichment of H3K18ac modification in the promotor regions of *Fcgr2b* and *Lyve1* was measured using ChIP-qPCR. “Input%” means the percent of chromatin enriched by H3K18ac in the total input chromatin. (**C**) The expression levels of FCGR2B and LYVE1 in rat liver with different doses of NaAsO_2_ treatment were measured using a Western blot, and the relative protein expression was quantitatively analyzed. In (**A**–**C**), the statistic “*F*” is derived from a one-way analysis of variance (one-way ANOVA). The notations “a, b, c” signify that there are significant differences (*p* value *<* 0.05) when compared with the three groups from 0.0 mg/kg to 5.0 mg/kg using Bonferroni correction, respectively. (**D**) The relationships between arsenic concentration in the liver (LAs), the enrichment of H3K18ac, expressions of *Fcgr2b* and *Lyve1*, the LSEC dedifferentiation indicator (CD31 expression), the HSC activation marker (α-SMA expression), the area of collagen deposition (CVF), and the content of Col Ⅰ in the liver were analyzed using correlation matrix. Blue squares and pink squares represent negative and positive correlations, respectively, with the darker color indicating the larger absolute value of the correlation coefficient.

H3K18ac is involved in cellular function through direct interaction with the gene promoter regions to regulate gene transcriptional activation [35]. To further reveal how H3K18ac regulates arsenic-induced LSEC dedifferentiation, the mRNA levels of specific genes that maintain the differentiation phenotype of LSECs were measured in the rat livers, including immunoglobulin gamma complex receptor IIB (*Fcgr2b*), lymphatic endothelial hyaluronic acid receptor 1(*Lyve1*), and liver sinusoidal scavenger receptors *Stabilin-1* and *Stabilin-2*. As shown in Figure 5A, the expression levels of *Fcgr2b* and *Lyve1* remarkably decreased with an increasing NaAsO_2_ treatment dose, whereas no response to the NaAsO_2_ treatment was observed for *Stabilin-1* and *Stabilin-2*. Next, the enrichment of H3K18ac in genes of *Fcgr2b* and *Lyve1* was measured to identify whether H3K18ac played a transcriptional regulation role in these two genes. The results showed that H3K18ac was enriched on eight promoter regions of the *Fcgr2b* and *Lyve1* genes, respectively, and the enrichments of H3K18ac on these fragments decreased in response to the NaAsO_2_ treatment (Figure 5B). Correspondingly, the mRNA and protein expression levels of *Fcgr2b* and *Lyve1* genes gradually decreased (Figure 5A,C). These results suggested that arsenic exposure could inhibit the transcription and protein expression of *Fcgr2b* and *Lyve1* genes by reducing H3K18ac enrichment in the genes’ promoter regions. Correlation matrix analysis comprehensively further found that the decreased enrichment of H3K18ac in *Fcgr2b* and *Lyve1* genes and the consequent repression of genes expression were not only associated with an increasing arsenic load in the livers but also significantly correlated with LSEC dedifferentiation, HSC activation, and the deposition of liver collagen fibers (Figure 5D). These observations suggested that arsenic exposure could lead to the dedifferentiation of LSECs by inhibiting the H3K18ac-dependent transcriptional activation of *Fcgr2b* and *Lyve1* genes, thereby promoting liver fibrosis.

## 4. Discussion

Chronic arsenic exposure could lead to progressive liver injury and subsequent irreversible outcomes, such as liver cirrhosis and liver cancer. Liver fibrosis is a reversible pathological stage in the progression of arsenic-induced chronic liver injury to cirrhosis and liver cancer, but the underlying mechanism remains unclear. Given that epigenetic modification is considered to be a promising option due to its reversibility, exploring the epigenetic mechanism of arsenic-induced liver fibrosis is helpful in providing new insights for the early prevention and treatment of arsenic-induced liver damage. This study revealed that the dedifferentiation of LSECs, the gatekeeper of the hepatic sinusoidal microenvironment, might be involved in arsenic-induced HSC activation and subsequent liver fibrosis. We also identified the role of H3K18ac in arsenic-induced LSEC dedifferentiation. Arsenic-suppressed H3K18ac could trigger the dedifferentiation of LSECs via the inhibiting transcriptional activation of *Fcgr2b* and *Lyve1*, which are key genes responsible for maintaining the differentiation phenotype of LSECs, thereby promoting liver fibrosis. This study uncovered the epigenetic mechanisms of arsenic-induced liver fibrosis from the novel perspective of LSEC dedifferentiation.

The pathological feature of liver fibrosis is the progressive accumulation of the extracellular matrix (ECM), leading to the destruction of the physiological structure of the liver [36]. Activated HSCs are the main source of ECM, as well as the hallmark of liver fibrosis [12]. The HSCs are located in Disse space and are surrounded by hepatocytes and LSECs. HSC activation depends on the interactions with surrounding cells within the hepatic sinusoid [37]. In chronic liver disease, persistent damage to the hepatocytes results in the continuous release of apoptotic and inflammatory factors. These stimuli not only directly activate the HSCs but also induce the recruitment and activation of lymphocytes and macrophages, which, in turn, promote the activation and differentiation of HSCs into extracellular matrix-producing fibroblasts through the production of pro-inflammatory and pro-fibroblast cytokines [38,39]. Differentiated LSECs are believed to be the maintainers of sinusoidal homeostasis due to their special fenestra structure and material exchange function. LSEC dedifferentiation promotes HSC activation by perturbing the crosstalk network between various cells, including hepatocytes, lymphocytes, macrophages, and HSCs [40]. Therefore, LSECs are known to be the gatekeepers of HSC activation, and in recent years, they have also been considered as an early intervention target for liver fibrosis [41]. Previous studies have reported that arsenic exposure can directly activate HSCs by triggering the key signaling pathways associated with HSC activation, such as TGF-β/sam and EGFR/ERK [42,43], and promote liver fibrosis by inducing abnormal crosstalk between the hepatocyte injury or inflammation [44], macrophage polarization [45], immune cell differentiation [46], and HSC activation [47]. For the first time, this study reveals the crosstalk association between LSEC dedifferentiation and arsenic-induced HSC activation and the role of this association in arsenic-induced liver fibrosis, providing new evidence about cell crosstalk to clarify arsenic-induced liver fibrosis.

Cell surface receptors are particularly important for maintaining differentiated phenotypes and the function of LSECs. This study found that arsenic exposure could induce LSEC dedifferentiation by inhibiting the expression of immunoglobulin gamma complex receptor IIB (FCGR2B) and lymphatic endothelial hyaluronic acid receptor 1 (LYVE-1). In the liver, FCGR2B and LYVE-1 are only expressed in LSECs, so they can be used to differentiate LSECs from other hepatic cell types. LYVE-1 is a marker for the maturation and differentiation of LSECs [48,49]. During liver regeneration, an increase in LYVE-1 expression favors the remodeling of the hepatic sinusoid, whereas a loss of LYVE-1 expression results in the destruction of the normal hepatic sinusoidal structure [50]. The decreased expression of LYVE-1 is associated with the loss of LSEC fenestration in inflamed or fibrotic livers, which is observed to be negatively correlated with liver fibrosis [51]. In the hepatic sinusoid, FCGR2B is responsible for mediating the clearance of small immune complexes (SIC) via LSECs, helping LSECs to function as the gatekeeper of the hepatic sinusoidal microenvironment [52]. Data from a non-alcoholic fatty liver inflammation biopsy specimen showed that a decreased FCGR2B expression level in LSECs was correlated with increased serum levels of collagen IV and hyaluronic acid and inversely correlated with the stage of fibrosis, suggesting that a decreased FCGR2B expression level promotes liver fibrosis by inhibiting LSECs’ clearance ability [53,54]. This evidence supports our findings that the reduced expression of FCGR2B and LYVE-1 is an important trigger for the arsenic-induced loss of differentiated phenotypes in LSECs. Additionally, our study further sheds light on the epigenetic mechanisms of arsenic-induced inhibition of FCGR2B and LYVE-1 expression that depend on H3K18ac regulation. At present, only the evidence from extrahepatic studies has elucidated the mechanisms of FCGR2B and LYVE-1 deficiency from the transcription factor pathways, such as the HIF-1α/PROX-1 and PI3K/AKT/mTOR pathways [55,56]. Our results provide new epigenetic evidence to understand the mechanism of LSEC dedifferentiation driven via the aberrant expression of cell surface receptors.

Growing evidence suggests that H3K18ac is involved in the regulation of various liver diseases. An aberrant increase in H3K18ac content was found to trigger acute hepatitis; the inhibitors of H3K18ac acetyltransferase could alleviate acute liver injury by reducing H3K18ac enrichment in the promoter region of the genes responsible for macrophage polarization [25]. Aberrant H3K18ac has also been shown to affect lipid oxidation in hepatocytes by disrupting the expression of FGF21, a glucose and lipid metabolism regulator [57]. Additionally, aberrant H3K18ac was also identified as a risk factor for poor survival outcomes in hepatocellular carcinoma cases [26]. This evidence suggests that H3K18ac may be an important epigenetic marker of different degrees and types of liver disease, as well as a target for risk monitoring and treatment. Previously, we identified the suppression of H3K18ac as an epigenetic marker in response to arsenic exposure and associated with increased risk of arsenic poisoning (the main manifestations of skin and liver damage) using a population study [23]. In an animal model of sub-chronic arsenic exposure, we demonstrated that H3K18ac might be involved in arsenic-induced early liver damage by modulating oxidative damage [24]. This study further uncovered the important role of H3K18ac-dependent LSEC dedifferentiation in arsenic-induced liver fibrosis. Taken together, consistent with the liver disease model, H3K18ac also plays a central regulatory role in the process of liver injury induced by environmental arsenic contamination.

Epigenetic modification is not only a sensitive biomarker of disease progression and prognosis but also a promising target for precision therapy due to its reversibility [58,59]. Emerging evidence suggests that epigenetic modification seems to be a novel mechanism to maintain LSEC differentiation and a new target for anti-fibrosis by correcting LSEC dedifferentiation. For example, microRNA-322/424 regulates LSEC dedifferentiation by targeting the CUL2/HIF-1α pathway to promote liver fibrosis, and correcting these aberrant microRNAs can alleviate liver fibrosis [60]. Similarly, the targeted maintenance of LncRNA Airn function can maintain the differentiation of LSECs through the KLF2-eNOS-sGC pathway, thereby inhibiting the activation of HSCs and preventing liver fibrosis [61]. Moreover, reducing the activity and expression levels of the histone deacetylase SIRT1 or knocking down histone acetyltransferases CBP/p300 in LSECs can induce LSEC dedifferentiation, ultimately leading to HSC activation, which can be rescued by restoring the expression and activity of these enzymes [62]. However, unlike the non-coding RNAs described above, the specific histone sites that regulate LSEC dedifferentiation are still unclear, which may hinder the exploration of precise pathological mechanisms and intervention targets. Our study identified H3K18ac as a specific histone site for the regulation of LSEC dedifferentiation and revealed that the underlying mechanism might be related to the repression of the H3K18ac-dependent transcription of cell differentiation maintenance genes. These findings provide epigenetic targets for precise intervention in LSEC dedifferentiation.

The following limitations remain in this study. Firstly, in this study, evidence from animal models revealed that the crosstalk between LSEC dedifferentiation and HSC activation might be the key mechanism of arsenic-induced liver fibrosis, but direct evidence and the underlying mechanisms of cell–cell interactions need to be explored further. Furthermore, based on the role of H3K18ac-dependent LSEC dedifferentiation in arsenic-induced liver fibrosis revealed in this study, more research is required to explore the possibility of H3K18ac as an intervention target to treat arsenic-induced LSEC dedifferentiation and liver fibrosis.

## 5. Conclusions

**Figure 6:**
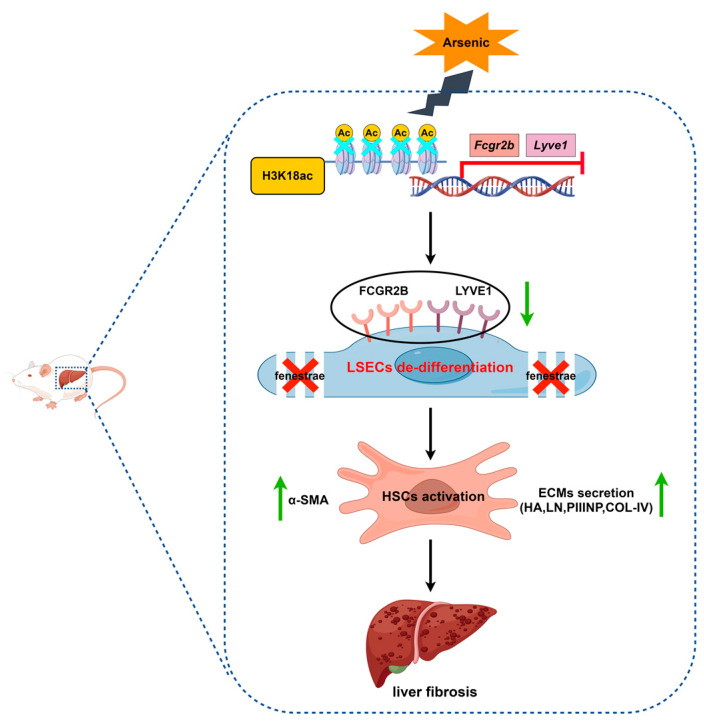
Arsenic-suppressed H3K18ac could trigger the dedifferentiation of LSECs by inhibiting transcriptional activation of *Fcgr2b* and *Lyve1*, which are key genes responsible for maintaining the differentiation phenotype of LSECs, thereby promoting HSC activation and liver fibrosis (The figure was made using Figdraw).

This study revealed that chronic arsenic exposure might induce HSC activation and subsequent liver fibrosis by triggering dedifferentiation of LSECs, the gatekeeper of the hepatic sinusoidal microenvironment. We also identified that arsenic-suppressed H3K18ac could play an important role in triggering LSEC dedifferentiation by inhibiting the transcriptional activation of *Fcgr2b* and *Lyve1*, which are the key genes responsible for maintaining the differentiation phenotype of LSECs (Figure 6). These findings uncover a novel mechanism of arsenic-induced liver fibrosis based on a new insight into epigenetically dependent LSEC differentiation.

## Data Availability

The data presented in this study are available upon request from the corresponding authors.

## Supplementary Materials

The following supporting information can be downloaded at: https://www.mdpi.com/article/10.3390/toxics11110928/s1, Table S1: The primers for ChIP-qPCR. Table S2: The primers for qPCR. Figure S1: The enrichment of IgG in promoters of *Fcgr2b* and *Lyve1* genes in rat liver. Figure S2: The relationships of arsenic concentration, HSCs activation, and collagen deposition in the liver. Figure S3: The associations of arsenic concentration, LSECs dedifferentiation, and the ECMs levels in the liver. Figure S4: The relationships of H3K18ac and the ECMs levels in the liver.
