# Supplementary material for: Suppressed Histone H3 Lysine 18 Acetylation Is Involved in Arsenic-Induced Liver Fibrosis in Rats by Triggering the Dedifferentiation of Liver Sinusoidal Endothelial Cells
Source: Toxics. 2023 Nov 13;11(11):928. doi: 10.3390/toxics11110928 (PMC10675694; doi:10.3390/toxics11110928)
Supplement: Supplementary file 1 [file toxics-11-00928-s001.zip › Supplemental.pdf]

Table S1 The primers for ChIP-qPCR.

Genes	Location (TSS)		Primer (5'-3')
Fcgr2b			
CHIP1	-2897~-2802	Forward	TCCCAAAGAAGAAAAAGCCAGAGC
		Reverse	AAGTTTCTTGGTCATGGTGGACTA
CHIP2	-1776~-1674	Forward	AGCAGGCAGGTCTCTGGATA
		Reverse	CTGCCCTCTACCACAAAGCA
CHIP3	-1246~-1087	Forward	AAGAGGCCTCAGAAAAGCTCC
		Reverse	TGCCACTGGTAAGGAACCAAC
CHIP4	-716~-553	Forward	CGGGTAGCCTGAAATGCTCA
		Reverse	TCCCCTGAAGTGCCTAGTGA
CHIP5	219~306	Forward	AACCAACGTGGCCTAGGAAG
		Reverse	AAGGCCGACCAGGTTGTTAC
CHIP6	1145~1284	Forward	CTGGCGAGAACTCCCAAGAA
		Reverse	CAGGAGCAAAGAAGATCCCGT
CHIP7	2109~2199	Forward	GAACACGTTAAGGGGACCCA
		Reverse	CAACTTGTCGTGTTGCCCAT
CHIP8	2779~2920	Forward	GGTGACCACATGGTGCATTC
		Reverse	TTTGATTCCCCGGCTTGTGG
Lyve1			
CHIP1	-2828~-2746	Forward	GCTCAGCTGTGGCAATTTGT
		Reverse	TGCAGGCCAGTTTTACTCCA
CHIP2	-1144~-1046	Forward	TCCAATCACCTGCCTCGAAC
		Reverse	CTAAGCAGCCGCCTACTCTC
CHIP3	-394~-314	Forward	GCCGTTCAGACCGCCTTTAT
		Reverse	ACTTAGGAGCAACCGAACGA
CHIP4	529~620	Forward	TGGGAATACGGGAGACTCGT
		Reverse	ATTTGGAGAGTCTGGCGGTG
CHIP5	1096~1231	Forward	CTGCTGAGGATGCCCTGAAA
		Reverse	GTTCTGGCATTGCAGTGGTG
CHIP6	1588~1738	Forward	TGAGCCACATCCCTAGCTCT
		Reverse	TCTGTACCTCTCCTCGCCTC
CHIP7	2403~2528	Forward	TACACGGCTGGAATGCCTTT
		Reverse	TTGACACGCTTCTCTGGGAC
CHIP8	3238~3339	Forward	CCATCAGGCAGTACAAGCGT
		Reverse	CCCTGGTTTGTGAGACTGGT

Table S2 The primers for qPCR.

Genes	Products length (bp)	Primer (5'-3')	
Fcgr2b	83	Forward	CACTGATGTGCGAAGGGAC
		Reverse	GGCTTGATGCCAGATGGA
Lyve1	154	Forward	GTTGAAAGTGGAGCAGCAT
		Reverse	TGTTTGTGAAAGGGAAGG
Stabilin-1	202	Forward	ACTCTACCTTCTGCCTATACCG
		Reverse	TAGCCGTCACCCACCTCA
Stabilin-2	174	Forward	GGCTCTTTACCAAGTCTACTC
		Reverse	TGTGGCTTTCTTCTCCCT
Gapdh	96	Forward	GGCTCTCTGCTCCTCCC
		Reverse	CCGTTCACACCGACCTT

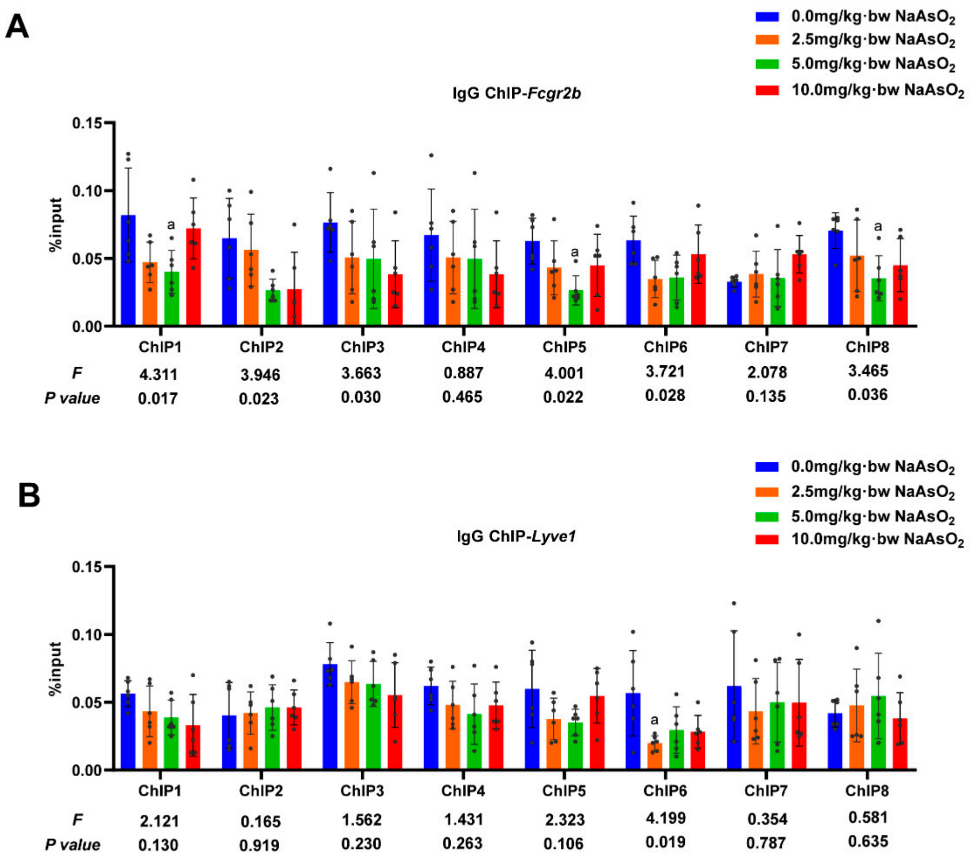

Fig.S1 The enrichment of IgG in promoters of *Fcgr2b* and *Lyve1* genes in rat liver. (A): The enrichment of IgG in promoters of *Fcgr2b* genes in rat liver; (B): The enrichment of IgG in promoters of *Lyve1* genes in rat liver. The statistic "*F*" is derived from a one-way analysis of variance (one-way ANOVAs). The notation "a" signifies that there are significant differences (P<0.05) when compared with the three groups from 0.0 mg/kg to 5.0 mg/kg using Bonferroni correction, respectively.

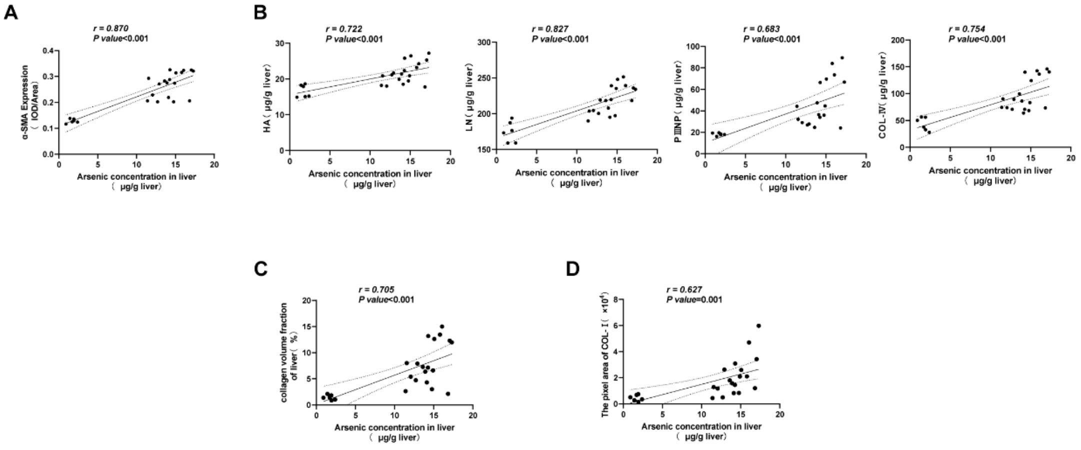

Fig.S2 The relationships of arsenic concentration, HSCs activation, and collagen deposition in the liver. The Pearson correlation analysis was used to assess the correlations between the arsenic concentration in rat liver with (A)the HSCs activation marker (α-SMA expression), (B)the ECMs levels (including hyaluronidase (HA), laminin (LN), procollagen III (PN IIIP) and collagen IV (COL-IV)), (C) the area of collagen deposition and (D)the content of Col Ⅰ in the liver.

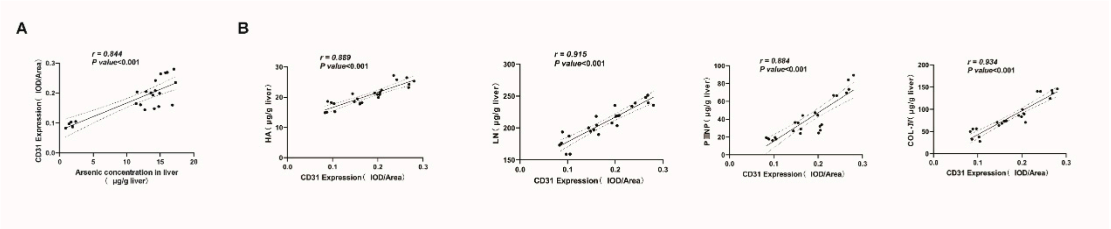

Fig.S3 The associations of arsenic concentration, LSECs dedifferentiation, and the ECMs levels in the liver. (A) The Pearson correlation analysis was used to assess the correlations between the arsenic concentration in rat liver and the LSECs dedifferentiation indicator (CD31 expression). (B) The Pearson correlation analysis was used to assess the associations of CD31 expression with the ECMs levels (including HA, LN, PN IIIP and COL-IV)

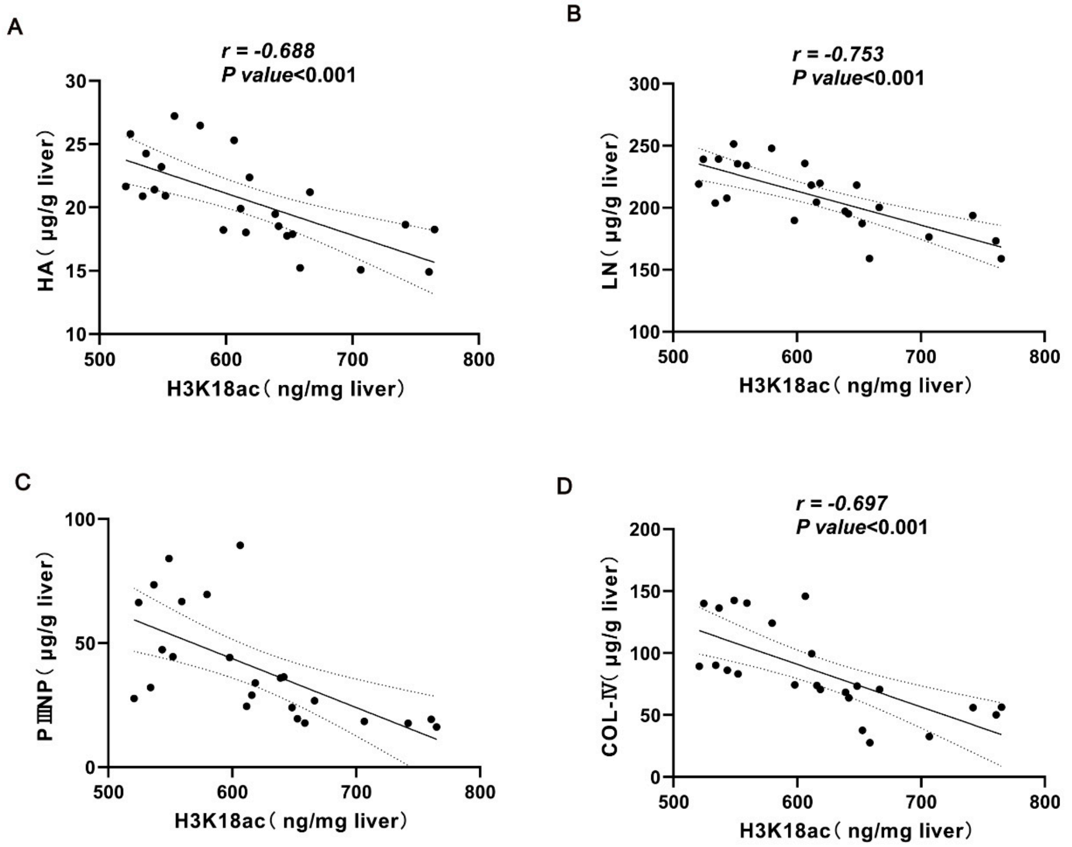

Fig.S4 The relationships of H3K18ac and the ECMs levels in the liver. The Pearson correlation analysis was used to assess the associations of the total level of H3K18ac modification in liver tissue with the ECMs levels, including (A)HA, (B)LN, (C)PIIINP and (D)COL-IV.
