# Supplementary figures and images for: Suppressed Histone H3 Lysine 18 Acetylation Is Involved in Arsenic-Induced Liver Fibrosis in Rats by Triggering the Dedifferentiation of Liver Sinusoidal Endothelial Cells
Source: Toxics. 2023 Nov 13;11(11):928. doi: 10.3390/toxics11110928 (PMC10675694; doi:10.3390/toxics11110928)

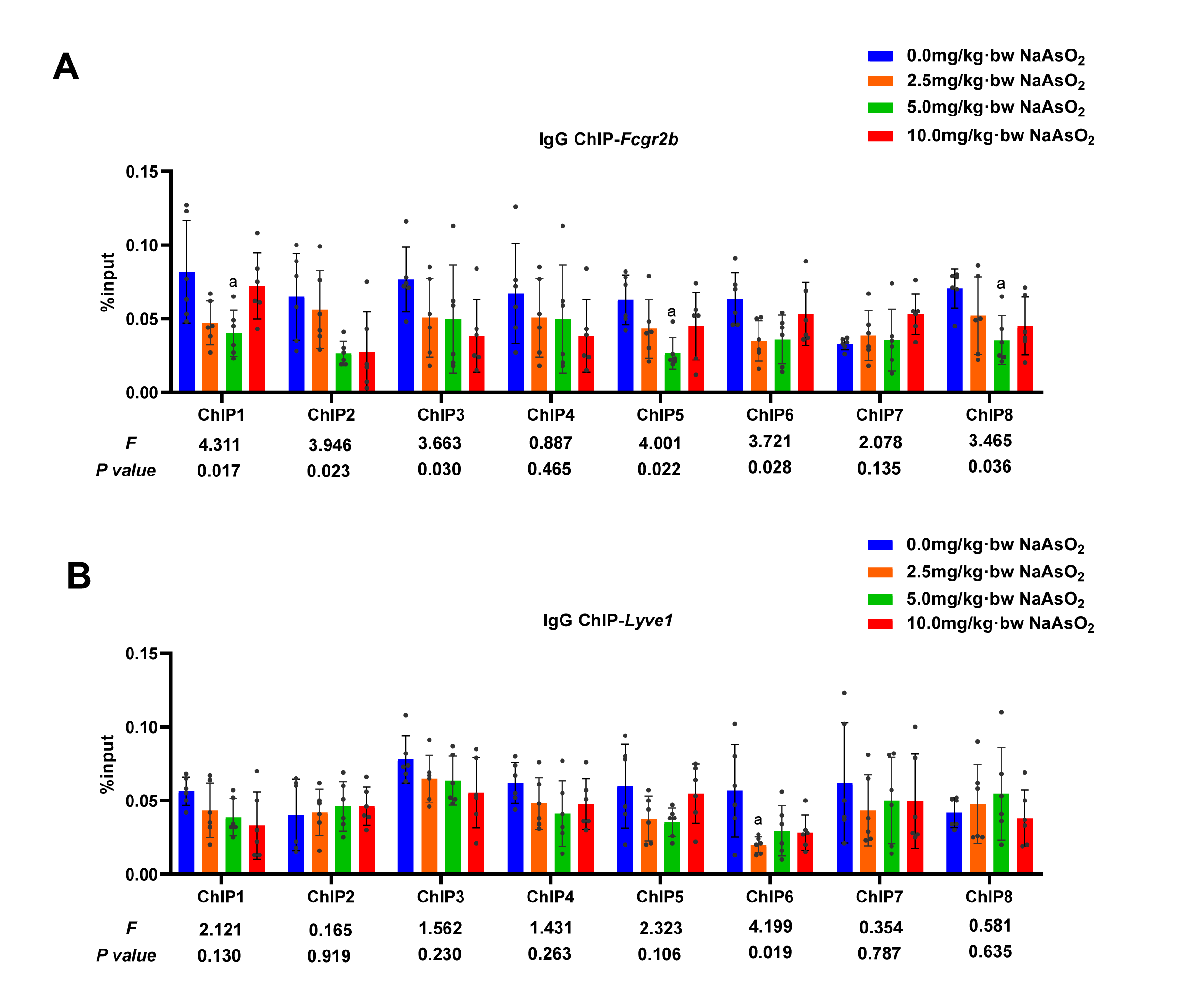

Supplement: Supplementary file 1 [file toxics-11-00928-s001.zip › Figure S1-01.tif]

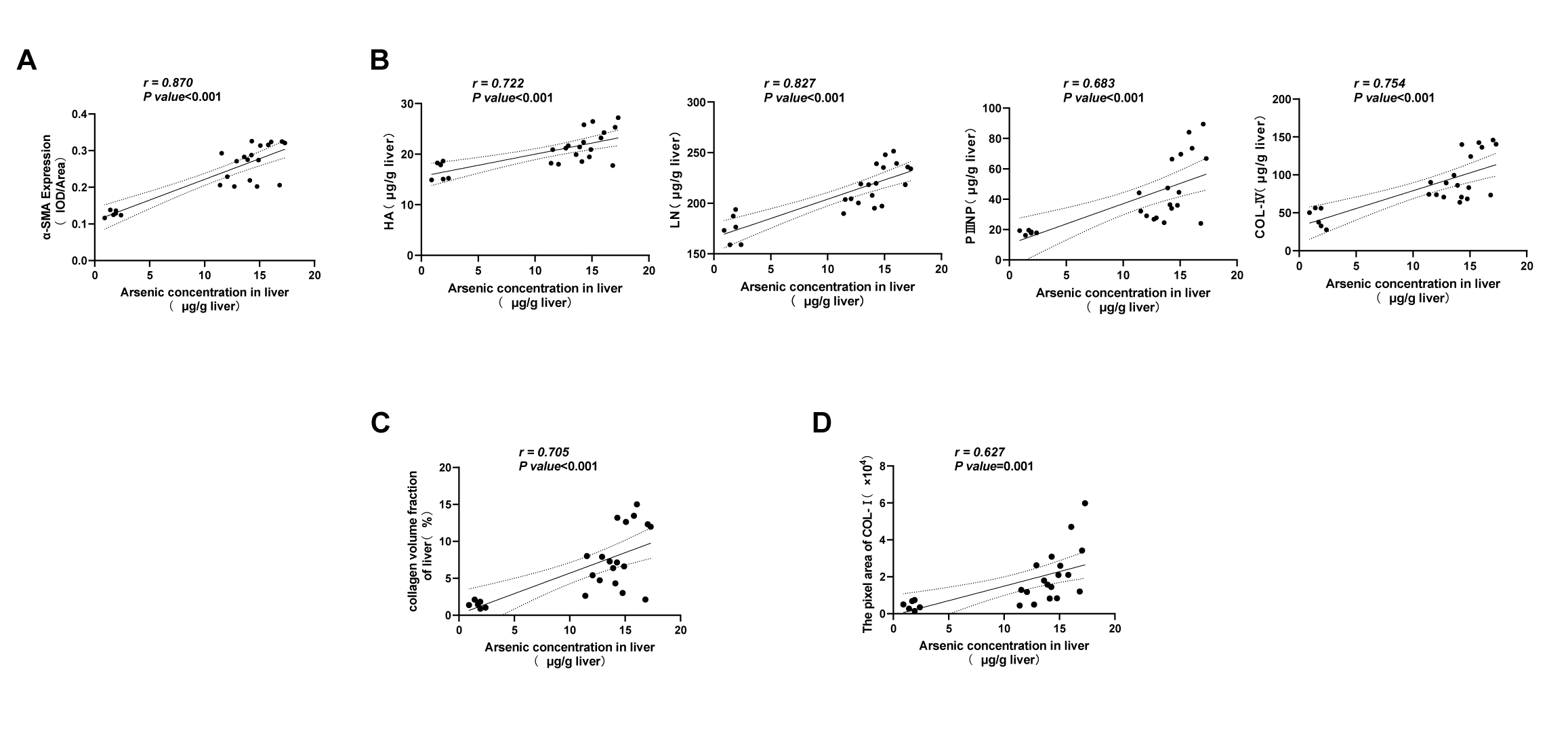

Supplement: Supplementary file 1 [file toxics-11-00928-s001.zip › Figure S2-01.tif]

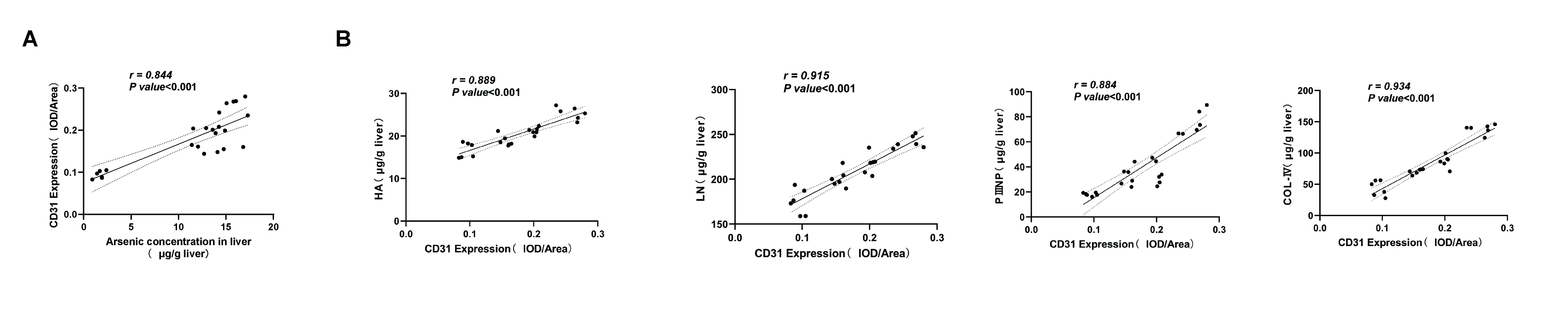

Supplement: Supplementary file 1 [file toxics-11-00928-s001.zip › Figure S3-01.tif]

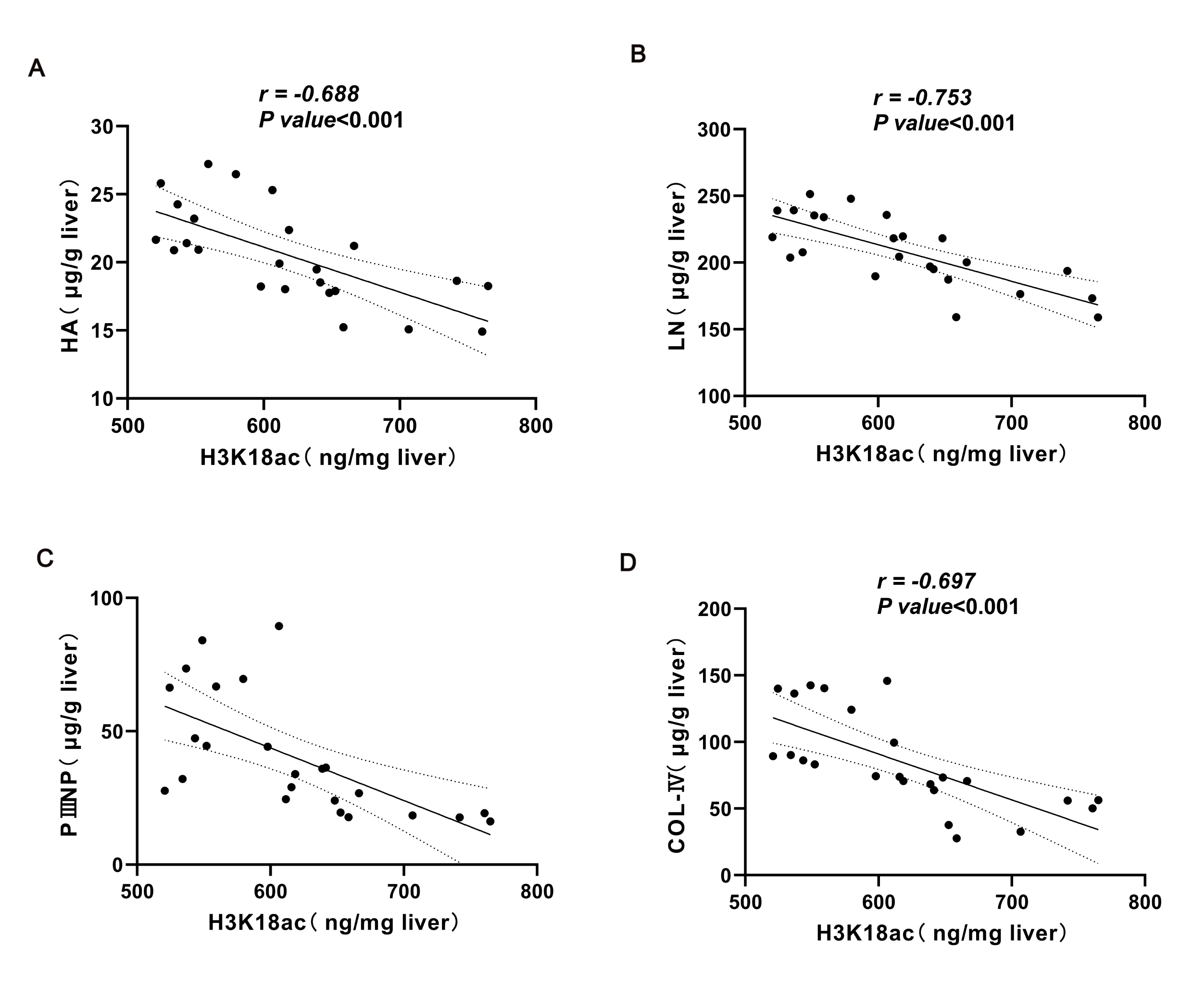

Supplement: Supplementary file 1 [file toxics-11-00928-s001.zip › Figure S4-01.tif]
